# An *Acanthamoeba* sp. containing two phylogenetically different bacterial endosymbionts

**DOI:** 10.1111/j.1462-2920.2007.01268.x

**Published:** 2007-06

**Authors:** Eva Heinz, Irina Kolarov, Christian Kästner, Elena R Toenshoff, Michael Wagner, Matthias Horn

**Affiliations:** Department of Microbial Ecology, Faculty of Life Sciences, University of Vienna A-1090 Vienna, Austria

## Abstract

Acanthamoebae are ubiquitous free-living amoebae and important predators of microbial communities. They frequently contain obligate intracellular bacterial symbionts, which show a worldwide distribution. All *Acanthamoeba* spp. described so far harboured no or only a single specific endosymbiont phylotype, and in some cases evidence for coevolution between the symbiotic bacteria and the amoeba host has been reported. In this study we have isolated and characterized an *Acanthamoeba* sp. (strain OEW1) showing a stable symbiotic relationship with two morphologically different endosymbionts. 16S rRNA sequence analysis assigned these symbionts to the candidate genus *Procabacter* (*Betaproteobacteria*) and the genus *Parachlamydia* (*Chlamydiae*) respectively. Fluorescence *in situ* hybridization and transmission electron microscopy confirmed the affiliation of the endosymbionts and showed their co-occurrence in the amoeba host cells and their intracellular location within separate compartments enclosed by host-derived membranes. Further analysis of this stable relationship should provide novel insights into the complex interactions of intracellular multiple-partner associations.

## Introduction

Acanthamoebae are free-living amoebae, which are ubiquitous and have been isolated from a wide variety of habitats ranging from natural sources like soil, salt water, fresh water and dust, to anthropogenic habitats like tap water, air-conditioning units and sewage systems (Rodriguez-Zaragoza, 1994; Marshall *et al*., 1997). Acanthamoebae are important predators feeding on various organisms like bacteria and fungi and hence control microbial communities (Rodriguez-Zaragoza, 1994). However, several bacteria have developed strategies to survive phagocytosis and are able to multiply within amoebae. Two different forms of such interactions can be distinguished. Some bacteria have evolved mechanisms to exploit the amoebae as a vessel for replication; in this case the relationship with the amoebae is only transient (Greub and Raoult, 2004). A well studied example is *Legionella pneumophila*, the causative agent of Legionnaire's disease, where intracellular replication within amoebae is considered an important step prior to the infection of humans (reviewed in Molmeret *et al*., 2005). Furthermore, the intracellular lifestyle protects the bacteria from the environment and enables them to survive in much harsher conditions. Being an important environmental reservoir of bacterial pathogens, free-living amoebae are also referred to as ‘Trojan horses’ of the microbial world (Barker and Brown, 1994). The second group of bacteria interacting with free-living amoebae are able to maintain a steady relationship with their hosts and are therefore called endosymbionts and apparently do not thrive outside of their hosts. Different evolutionary lineages of bacterial endosymbionts of acanthamoebae have been identified to date, which are found within the *Alphaproteobacteria*, the *Betaproteobacteria*, the *Bacteroidetes* and the *Chlamydiae* (reviewed in Horn and Wagner, 2004). Although very distantly related, these four groups were found in amoeba isolates from a wide variety of habitats, showing a worldwide distribution (Horn and Wagner, 2004). At least for one alphaproteobacterial group of *Acanthamoeba* symbionts, coevolution between both symbiosis partners seems highly likely, further supporting the stability of these relationships over evolutionary time periods (Beier *et al*., 2002).

All symbioses between bacteria and acanthamoebae studied so far involved only two partners, intracellular bacteria of a single phylotype and the amoeba host. Here we report on the analysis of *Acanthamoeba* sp. OEW1 isolated from a saline lake in Austria. We could show that *Acanthamoeba* sp. OEW1 formed a stable symbiotic relationship with two phylogenetically different endosymbionts residing within each amoeba host cell.

## Results and discussion

### Isolation of *Acanthamoeba* sp. OEW1

Sediment samples collected at the eastern part of lake ‘Wörthenlacke’ (eastern Austria) showing a high salinity and a pH of 8.3 (Metz and Forró, 1989) were investigated for the presence of amoebae, which were initially isolated on non-nutrient agar plates seeded with live or heat-inactivated *Escherichia coli* (Page, 1988). After repeated transfer to fresh plates, amoebae were transferred to liquid medium (containing 30 g l^−1^ trypticase soy broth, 10 g l^−1^ yeast extract, pH 7.3) and subsequently maintained as axenic culture. The isolated amoebae showed a large single nucleus with a prominent nucleolus, needle-like pseudopodia, and a large contractile vacuole, characteristic features of the genus *Acanthamoeba* (Page, 1988), and was therefore designated *Acanthamoeba* sp. strain OEW1 (deposited at the American Type Culture Collection ATCC under accession number PRA-220). Analysis of the 18S rRNA gene of *Acanthamoeba* sp. strain OEW1 confirmed the morphology-based assignment and showed that it belongs to the 18S rRNA gene sequence type 4 (data not shown; Stothard *et al*., 1998). Epifluorescence microscopy and staining with the general DNA dye 4,6-diamidino-2-phenylindoldihydrochlorid (DAPI) readily visualized two different morphotypes of bacteria, one rod-shaped and one coccoid form, co-occurring within the same amoeba cells (data not shown). All amoebae in the culture were simultaneously infected by both endosymbionts, and the amoeba culture as well as the presence of both symbionts remained stable over several months.

### The two endosymbionts of *Acanthamoeba* sp. OEW1

**Fig. 1:**
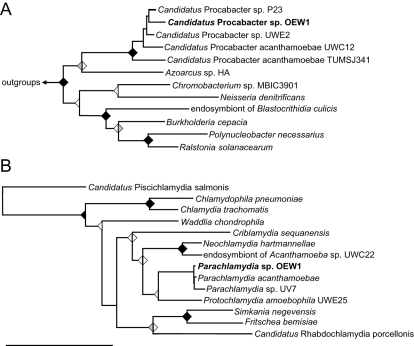
Phylogenetic affiliation of the two endosymbionts of *Acanthamoeba* sp. OEW1, ‘*Cand*. Procabacter sp. OEW1’ (A) and *Parachlamydia* sp. OEW1 (B) based on 16S rRNA sequence analysis. Tree calculations were performed using the maximum likelihood (AxML), neighbour joining and maximum parsimony algorithms implemented in the ARB program package (Ludwig *et al*., 2004). Maximum likelihood trees are shown, neighbour joining (left triangle) and maximum parsimony (right triangle) bootstrap values (1000 re-samplings) are indicated for supported nodes; black triangles, bootstrap values > 90%; grey triangles, bootstrap values ≥ 75%; white triangles, bootstrap values < 75%. Bar represents 10% estimated evolutionary distance.

In order to identify the bacterial endosymbionts of *Acanthamoeba* sp. OEW1, DNA was extracted from amoeba cultures with the FastDNA-Kit (MP Biomedicals, Heidelberg, Germany), and the 16S rRNA genes were amplified using primers 616V (5′-AGAGTTTGATYMTGGCTC-3′) and 630R (5′-CAKAAAGGAGGTGATCC-3′) at an annealing temperature of 52°C. These primers target most bacteria (Loy *et al*., 2002), but show mismatches with the *Chlamydiae*, some of which are well known as symbionts of amoebae (Amann *et al*., 1997; Fritsche *et al*., 2000; Horn *et al*., 2000). Therefore, we additionally used primers panF (5′-CGTGGATGAGGCATGCRAGTCG-3′) and panR (5′-GTCATCRGCCYYACCTTVSRCRYYTCT-3′), targeting most chlamydiae (Corsaro *et al*., 2002), in a separate polymerase chain reaction (PCR) at an annealing temperature of 65°C. Amplificates of the expected size were obtained with both PCR approaches and could be sequenced directly. Comparative analysis of the retrieved near-full-length 16S rRNA sequences revealed that the two symbionts were affiliated with different bacterial lineages, the *Betaproteobacteria* and the *Chlamydiae* respectively. The betaproteobacterial endosymbiont showed closest 16S rRNA sequence homology to the amoeba symbiont ‘*Candidatus* Procabacter sp. P23’ and was designated ‘*Candidatus* Procabacter sp. OEW1’ (98.2%; Horn *et al*., 2002). The chlamydial endosymbiont was most similar to *Parachlamydia acanthamoebae* Bn_9_ (99.8%; Amann *et al*., 1997) and was named *Parachlamydia* sp. OEW1. Phylogenetic analysis consistently confirmed the similarity-inferred affiliation of both symbionts (Fig. 1).

**Fig. 2:**
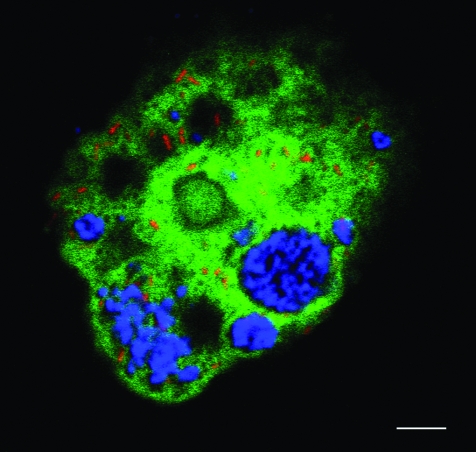
*In situ* identification of the two endosymbionts of *Acanthamoeba* sp. OEW1, *Parachlamydia* sp. OEW1 and ‘*Cand*. Procabacter sp. OEW1’. The probes used in this analysis were Proca-438 directly labelled with the hydrophilic sulfoindocyanine fluorescent dye Cy3 specific for ‘*Candidatus* Procabacter acanthamoebae’ (Horn *et al*., 2002), Bn9-658 labelled with Cy5 targeting a subgroup of the *Parachlamydiaceae* (Amann *et al*., 1997) and EUK-516 labelled with 5(6)-carboxyfluorescein-*N*-hydroxy-succinimide (FLUOS) targeting most members of the *Eukarya* (Amann *et al*., 1990). To ensure specificity hybridization was performed with 20% formamide in the hybridization buffer and corresponding salt concentration in the washing buffer. For further details on oligonucleotide probes, see probeBase at http://www.microbial-ecology.net/probebase (Loy *et al*., 2007). The overlay of the FISH micrographs, illustrating Cy3 in red, Cy5 in blue, and FLUOS in green, demonstrates the intracellular location of both endosymbionts within the same amoeba host cell. Fluorescence *in situ* hybridization was performed as described previously (Manz *et al*., 1992; Horn *et al*., 2001) and examined by a confocal laser scanning microscope (LSM 510 Meta, Carl Zeiss, Jena, Germany). All experiments were performed at least three times and yielded consistent results. Intervals of at least 1 week separated individual experiments. Bar, 5 μm.

The identity and intracellular location of both bacterial symbionts could be further demonstrated by fluorescence *in situ* hybridization (FISH) in combination with laser scanning microscopy. Both endosymbionts were detected with probes specific for the two groups of endosymbionts (for further details see Fig. 2), demonstrating that all amoebal cells within the population harboured both endosymbionts. All detected bacteria could be visualized with either of the two endosymbiont-specific probes showing the absence of further, not recognized bacteria within the amoebae (data not shown). *Parachlamydia* sp. OEW1 resided within one or few large inclusions containing numerous bacteria, whereas the ‘*Cand*. Procabacter sp. OEW1’ was spread evenly throughout the host cell (Fig. 2).

**Fig. 3:**
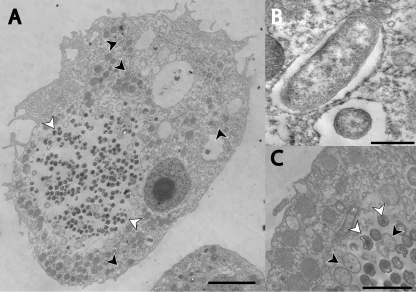
Ultrastructure of *Acanthamoeba* sp. OEW1 and its endosymbionts, *Parachlamydia* sp. OEW1 and ‘*Cand*. Procabacter sp. OEW1’. A. Overview of one amoeba trophozoite containing numerous *Parachlamydia* sp. OEW1 in a large inclusion (white arrowheads) and few rod-shaped ‘*Cand*. Procabacter sp. OEW1’ (black arrowheads). Bar, 5 μm. B. Close-up on ‘*Cand*. Procabacter sp. OEW1’ enclosed by a host-derived membrane; a cross and a longitudinal section is depicted. Bar, 0.5 μm. C. Close-up on a *Parachlamydia* sp. OEW1 inclusion. Reticulate and elementary bodies are readily recognized (black and white arrowheads respectively). Bar, 2 μm. Samples for electron microscopy were fixed in 2.5% glutaraldehyde for 1 h at room temperature, post-fixed in 2% osmium tetroxide for 1 h at room temperature, prestained with 2% aqueous uranyl acetate, dehydrated with an ascending ethanol series and embedded in Low Viscosity Resin (Agar Scientific, UK). After polymerization for 16 h at 65°C, samples were cut on an ultramicrotome (Reichert Ultracut E) with a glass-knife and stained with 1% uranyl acetate for 4 min and 0.3% lead citrate for 2 min. Analysis was performed on a transmission electron microscope (TEM 902, Carl Zeiss, Jena, Germany). Cultures were embedded in five separate experiments, and a total of approximately 50 amoebal cells were examined closely and showed consistent results. Intervals of at least 1 week separated individual experiments.

Transmission electron microscopy was performed to get further insights into the ultrastructure of this symbiosis (for further details see Fig. 3). Both bacterial symbionts showed Gram-negative-type cell walls and were located within multiple separate compartments enclosed by host-derived membranes. *Parachlamydia* sp. OEW1 formed large inclusions containing numerous bacteria, which is a typical feature of members of the *Chlamydiae*. Within the inclusions, the characteristic stages of the biphasic chlamydial developmental cycle, elementary bodies and reticulate bodies dividing by binary fission, could be observed (Fig. 3; Moulder, 1991; Hackstadt *et al*., 1997; Fritsche *et al*., 2000). ‘*Cand*. Procabacter sp. OEW1’ resided within single cell inclusions, i.e. each symbiont was separately surrounded by a host-derived membrane. This seems remarkable as ‘*Cand*. Procabacter spp.’ have not been observed enclosed in host vacuoles before (Horn *et al*., 2002), indicating significant differences in lifestyle between closely related bacterial symbionts sharing the same group of host organisms.

### Multiple-partner associations

Bacterial symbionts of free-living amoebae have been known for a long time (Proca-Ciobanu *et al*., 1975; Fritsche *et al*., 1993), but have only recently been characterized on a molecular level (reviewed in Greub and Raoult, 2004; Horn and Wagner, 2004). A common characteristic of all *Acanthamoeba* isolates studied so far was the presence of only a single symbiont phylotype per amoeba host cell. *Acanthamoeba* sp. OEW1 is thus the first *Acanthamoeba* isolate containing two phylogenetically different bacterial symbionts that form a stable relationship with their host. Descriptions of multiple-partner associations are rather common in insects (Baumann, 2005), but have otherwise only been reported in distantly related organisms such as oligochaetes or molluscs (Distel *et al*., 1995; Dubilier *et al*., 2001). Among protists, there is only one report suggesting the occurrence of two different symbiont populations within the free-living amoeba *Naegleria clarki* N_DMLG (Walochnik *et al*., 2005), and the presence of different symbionts within a single host cell has been observed on several occasions in ciliates (Fokin, 2004). However, those symbionts still await their molecular identification.

Multiple-partner associations, in particular those where different intracellular symbionts share the same host cell, pose a number of challenges to all partners. The intracellular symbionts have to compete for nutrients possibly derived from the host cell while all interactions between the symbiosis partners need to be fine-tuned to ensure the stability of the relationship. Consistently, recent studies of multiple-partner associations including genomic methods showed complex adaptations, mainly regarding metabolic potential, of the symbionts as well as the host (Woyke *et al*., 2006; Wu *et al*., 2006). This raises the question whether the two amoebal symbionts described in this study have coevolved complementary metabolic abilities in dependence on each other. There might also exist a possibly mutual dependence between the host cell and one or both of its symbionts, potentially reinforcing a prolonged coevolution. However, to date virtually nothing is known about the interaction between ‘*Cand*. Procabacter’-related symbionts and their amoeba host. For the *Parachlamydia*-related symbiont *Protochlamydia amoebophila* UWE25, genomic and biochemical analysis demonstrated an intimate connection of symbiont and host nucleotide pools by means of specialized bacterial nucleotide transport proteins – a mechanism that could also be of importance in the symbiosis between *Acanthamoeba* sp. OEW1 and its bacterial symbionts (Haferkamp *et al*., 2004; 2006; Horn *et al*., 2004; Schmitz-Esser *et al*., 2004; Collingro *et al*., 2005).

### Lateral gene transfer between intracellular bacteria

As a consequence of their lifestyle obligate intracellular bacteria have only few opportunities to acquire genes from other microorganisms. Nevertheless, there is evidence for lateral gene transfer between chlamydiae and other microorganisms (Read *et al*., 2000; Schmitz-Esser *et al*., 2004; Gupta and Griffiths, 2006) and between different *Chlamydia trachomatis* strains (Demars *et al*., 2007). Furthermore, the genome of the amoeba symbiont *P. amoebophila* encodes a type 4 secretion system, which seems to have been acquired by lateral gene transfer (Greub *et al*., 2004; Horn *et al*., 2004; Collingro *et al*., 2005). Interestingly, highly similar genes were also found in the genome of *Rickettsia bellii*, obligate intracellular bacterial pathogens only distantly related to the *Chlamydiae* (Ogata *et al*., 2006). Phylogenetic analysis strongly indicated a common origin of these genes, possibly transferred in an early state of evolution when chlamydial and rickettsial ancestors might have shared the same amoeba host cell. This would be consistent with the unexpectedly large number of proteins encoded in the *R. bellii* genome showing highest homologies to other intracellular bacteria that can thrive in amoebae (Ogata *et al*., 2006). Free-living amoebae might thus represent hot spots for lateral gene transfer between intracellular bacteria. The identification of an *Acanthamoeba* sp. containing two phylogenetically different bacterial symbionts reported in this study lends additional support for this possible role of these protozoa. Further analysis of this trilateral symbiotic relationship could provide new insights into the adaptations leading to stable intracellular multi-partner associations and into the (co)evolution of organisms living in close proximity within a protected environment.

Nucleotide sequences reported in this study have been deposited at EMBL/GenBank/DDBJ under accession numbers AM412762 (partial 18S rRNA gene sequence of *Acanthamoeba* sp. OEW1), AM412760 (16S rRNA gene sequence of *Parachlamydia* sp. OEW1) and AM412761 (16S rRNA gene sequence of ‘*Cand*. Procabacter sp. OEW1’).
